# Mucosal Melanoma: Pathological Evolution, Pathway Dependency and Targeted Therapy

**DOI:** 10.3389/fonc.2021.702287

**Published:** 2021-07-19

**Authors:** Yanni Ma, Ronghui Xia, Xuhui Ma, Robert L. Judson-Torres, Hanlin Zeng

**Affiliations:** ^1^ Department of Oncology, Ninth People’s Hospital, Shanghai Jiaotong University School of Medicine, Shanghai, China; ^2^ Shanghai Institute of Precision Medicine, Shanghai, China; ^3^ Department of Oral Pathology, Ninth People’s Hospital, Shanghai Jiaotong University School of Medicine, Shanghai, China; ^4^ Department of Oral & Maxillofacial - Head and Neck Oncology, Ninth People’s Hospital, Shanghai Jiao Tong University School of Medicine, Shanghai, China; ^5^ Department of Dermatology, University of Utah, Salt Lake City, UT, United States; ^6^ Huntsman Cancer Institute, Salt Lake City, UT, United States

**Keywords:** mucosal melanoma, mucosal melanocytes, melanocytic lesions, mutations, signaling dependency, targeted therapy

## Abstract

Mucosal melanoma (MM) is a rare melanoma subtype that originates from melanocytes within sun-protected mucous membranes. Compared with cutaneous melanoma (CM), MM has worse prognosis and lacks effective treatment options. Moreover, the endogenous or exogenous risk factors that influence mucosal melanocyte transformation, as well as the identity of MM precursor lesions, are ambiguous. Consequently, there remains a lack of molecular markers that can be used for early diagnosis, and therefore better management, of MM. In this review, we first summarize the main functions of mucosal melanocytes. Then, using oral mucosal melanoma (OMM) as a model, we discuss the distinct pathologic stages from benign mucosal melanocytes to metastatic MM, mapping the possible evolutionary trajectories that correspond to MM initiation and progression. We highlight key areas of ambiguity during the genetic evolution of MM from its benign lesions, and the resolution of which could aid in the discovery of new biomarkers for MM detection and diagnosis. We outline the key pathways that are altered in MM, including the MAPK pathway, the PI3K/AKT pathway, cell cycle regulation, telomere maintenance, and the RNA maturation process, and discuss targeted therapy strategies for MM currently in use or under investigation.

## Introduction

Melanoma develops due to the unchecked proliferation of melanocytes, which are responsible for the production of pigment. About 90% of melanoma cases are cutaneous melanoma (CM) mainly induced by exposure to ultraviolet (UV) light (1). Non-cutaneous subtypes include uveal melanoma (UM) and mucosal melanoma (MM). MM is a rare type of melanoma that presents on mucosal surfaces of cavities within the body, including the oral, nasal, anorectal, genitourinary, and vulvovaginal region (2). Although MM makes up approximately 1% of all cases of melanoma, it is one of the most aggressive subtypes, and thus exhibits a worse prognosis compared with the common CM (3, 4). Based on a retrospective study, the 5-year survival rate of MM, considering all stages at the time of diagnosis, is 10-20% when compared to 93% for CM (4–6).

There are several possible reasons for this worse prognosis in MM: 1) Both the biology of mucosal melanocytes as well as the risk factors that are related to MM incidence are poorly understood. Exposure to UV is a well-established risk factor for CM but the mutagens that contribute to the development of MM remain unknown. According to epidemiological studies, smoking, ill-fitting dentures, and ingested/inhaled carcinogens such as tobacco and formaldehyde are regarded as potential causative factors for oral and sinonasal mucosal melanoma (2, 7), while chronic inflammatory disease, viral infections as well as chemical irritants are thought to be implicated in vulvar mucosal melanoma and human immunodeficiency virus (HIV) is associated with anorectal mucosal melanoma (2). However, the contributions and mechanisms of the aforementioned factors to MM initiation or progression are not clearly defined. 2) The evolution of MM from precursor lesions is poorly understood. CM is associated with different types of precursor lesions, including benign melanocytic nevi commonly associated with the *BRAF V600E* mutation and dysplastic nevi associated *NRAS* alterations and *TERT* promoter mutations (8). CM can evolve from these benign lesions following additional mutations that drive tumor invasion and metastasis such as loss of *CDKN2A*, *PTEN*, or *TP53 (* 9). Characterization of the morphology and molecular landscape of precursors compared to early melanoma has provided candidate molecular biomarkers for early diagnosis in CM (10–13). However, although several forms of mucosal melanocytic benign lesions are reported, there is still a lack of defined MM precursor lesions, leading to a weak understanding of the evolutionary trajectory of MM despite molecular profiles unveiled by recent whole-genome sequencing data (14–16). 3) MM has more diverse mutation patterns with fewer targetable mutations compared to CM. According to the most frequent and mutually exclusive mutations, CM is mainly classified into 4 genomic subtypes: *BRAF*(52%), *RAS*(31%), *NF1*(14%), and a small portion of triple wild-type (17, 18). Hence, co-targeting BRAF and MEK have been proved to achieve a significant response rate for *BRAF V600* mutated CM patients in clinical management (19, 20). In contrast, MM has more diverse mutation patterns, with less than 20% of *BRAFV600E* mutations (16), followed by the majority of mutations that are scattered and difficult to target, including *NRAS*, *NF1*, *KIT, SF3B1*, and *SPRED1* (21).

Our goals in conducting this review were to: 1) Summarize the types of mucosal melanocytic benign lesions, aiming to find possible genetic and pathological evolutional patterns from benign mucosal melanocytes lesions to malignant tumors; 2) Discuss the main driver mutations and pathways in MM; and 3) Outline the options of targeted treatment for MM in clinical use or under clinical trials.

## Biological Functions of Melanocytes

Melanocytes are neural crest-derived cells that migrate to specific anatomic locations - including skin, eyes, leptomeninges, and mucous membrane - during development. Cutaneous melanocytes have two final destinations: hair follicles and the basal cell layer of epithelium where they conduct their main biological function of melanin production (22, 23). Melanin is a natural pigment in skin that absorbs UV radiation and scavenges cytotoxic free radicals generated from sunlight exposure (23, 24). Synthesized melanin is secreted to the nearby keratinocytes under solar stimulation and protects the genome of keratinocytes from sun damage (25).

In addition to residing in the skin, melanocytes also dwell in many sun-shielded mucosal tissues like respiratory (oral, nasal, pharynx, larynx, and upper esophagus), intestinal, urogenital, and rectal tracts (2, 24, 26–28). As melanocytes located in mucous membranes are not usually directly exposed to sunlight, it is unlikely that photoprotection is the primary and definitive function of mucosal melanocytes. It was hypothesized that melanocytes localized to mucosal tissues due to errors of migration from the neural crest during embryogenesis (6, 26), but recent evidence suggests that mucosal melanocytes might have biological functions besides pigment production. Specifically, since mucosa plays an important role in the innate immune defense system, it is speculated that mucosal melanocytes are also equipped with immunogenic functions (23, 29).

It is reported that melanin has strong toxin binding properties that can neutralize toxins produced by bacteria (30). Meanwhile, aromatic precursors, including quinone and semiquinone intermediates generated during the melanization cascade, can disrupt the lipid bilayer of cell membranes of microorganisms and mediate an anti-bacterial effect (31, 32). The strong binding capacity of melanin is probably due to its specific graphite-like lamellar structure in which four to eight monomers are covalently bound to form a porphyrin-like system (33). As a result, melanin is able to interact with aromatic metabolites or compositions of microorganisms through hydrogen bonds or π-π interactions (34, 35). Another explanation for the anti-bacterial properties of melanin and its intermediates is that they contain high levels of redox-active catechol groups, which can produce reactive oxygen species under light and water stimulation (36). However, since the mucosal melanocytes are in a dark environment with marginal melanin production, it remains unknown whether the pigment levels in mucosal regions are sufficient for antimicrobial effects.

In addition to the anti-bacterial properties of melanin, melanocytes can also participate in the intrinsic and acquired immune system. On the one hand, melanocytes can participate in innate immunity since they are found to express Toll-like receptors, indicating melanocytes can recognize pathogen-associated molecular patterns present in microbes (37, 38). Once being recognized, bacteria and fungi can be engulfed by melanocytes – a phenomenon that has been observed under the microscope - before undergoing possible degradation pathways by lysosome hydrolytic enzymes contained in melanosomes (39, 40). On the other hand, melanocytes may be a component of acquired immunity. Melanocytes have been reported to express MHC class II loaded with mycobacterial peptides (41), suggesting that melanocytes may function as antigen-presenting cells and subsequently activate CD4^+^ T cells proliferation (42). Although the phagocytotic functions of melanocytes have been observed, the activation of T cells through antigen presented by melanocytes, for instance, should be further verified by investigating the expression of CD86, CD80, or other markers of antigen-presenting cells on the surface of melanocytes. Since melanocytes have the capacity to produce a variety of cytokines, including interleukins and interferons which may be involved in the regulation of the activity of neighboring immune cells under stimulations of exogenous nucleic acids (43–47), the hypothesis that melanocytes activate T cells through secreting specific cytokines, instead of acting as antigen-presenting cells, must also be tested. These collective observations suggest melanocytes likely participate to some extent in the immune defense of the body, but their precise immunological roles in the innate or adaptive immune system need to be dissected in further studies.

## Mucosal Melanocytic Benign Lesions

**Figure 1:**
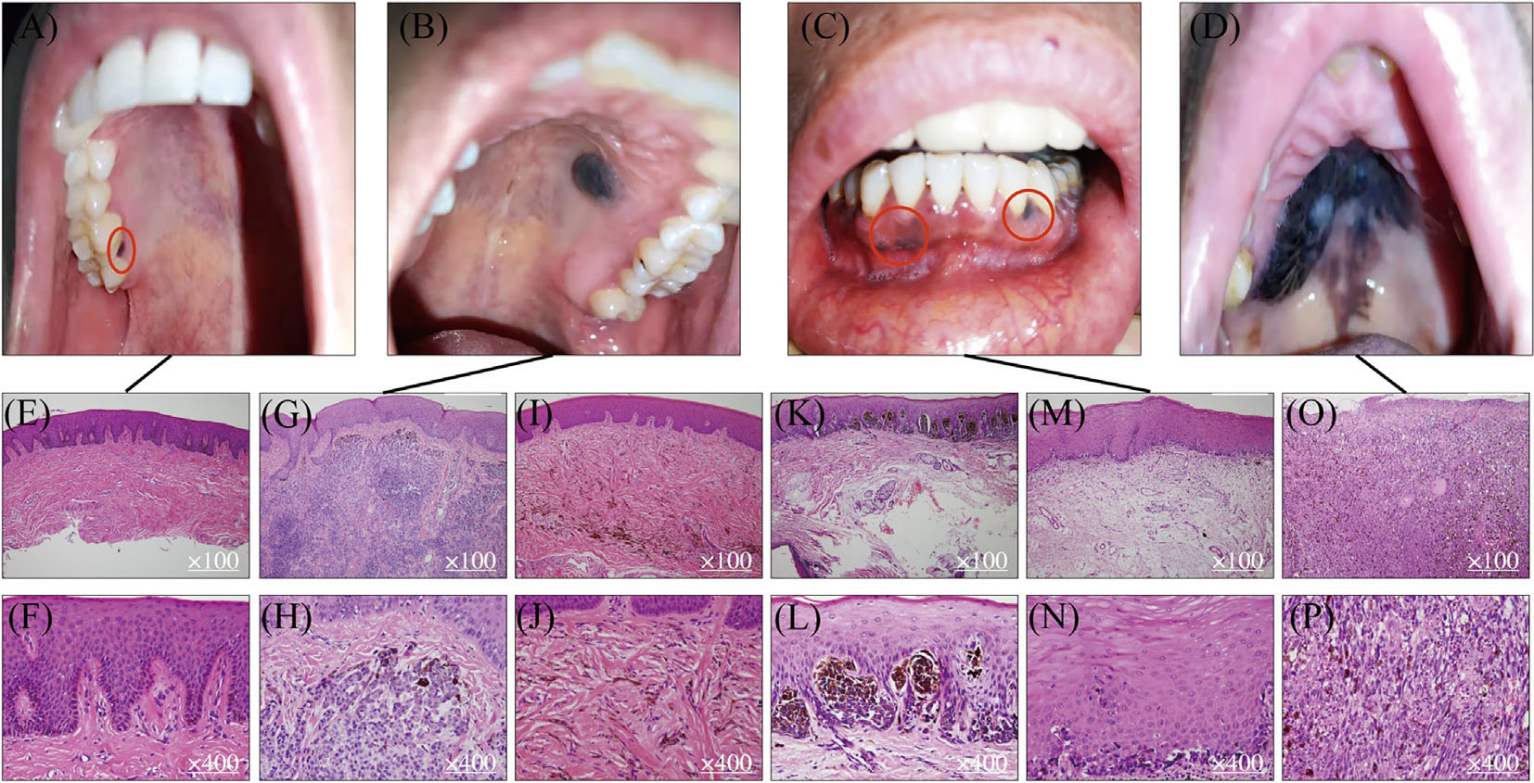
Mucosal melanocytic benign lesions and malignant OMM. Benign hyperpigmented lesions **(A, B)** and malignant OMM in situ **(C, D)**. Benign macule in gingiva **(A)** and its HE staining pictures **(E, F)**. Benign intramucosal nevus on the hard palate **(B)** and its HE staining pictures **(G, H)**. HE staining of blue nevus **(I, J)**. HE staining of junctional nevus **(K, L)**. Lentigo maligma melanoma on mandibular gingiva **(C)** and its HE staining figures **(M, N)**. Ulcerated malignant MM on the hard palate **(D)** and its HE staining pictures **(O, P)**.

Studies in the pathologic evolution of CM have shown that invasive melanomas can evolve from a variety of benign and intermediate pathological stages including benign nevus, dysplastic nevus, and malignant tumor in situ (8, 9). Melanocytic nevi are benign lesions requiring no further treatment, while atypical melanocytic hyperplasia or atypical nevi are regarded as either indeterminant or premalignant lesions that warrant careful clinical management and long-term follow-up for patients. In contrast, there is no clear definition and characterization of precursor lesions of MM despite the fact that multiple mucosal melanocytic benign lesions are observed and documented in the clinic (**Figures 1A, B**). Using OMM as the most well-studied example, **Table 1** summarizes several benign pigmented lesions including macule, nevus, and melanocanthoma with their specific pathological characteristics.

**Table 1:** Comparison of benign lesions and malignant oral mucosal melanoma.

	Macule	Nevi	Melanoacanthoma	OMM
Prevalence in melanocytic lesions	62% (48, 49)	15% (48, 49)	0.8% (48, 49)	0.7% (48, 49)
Color	Gray to brown to black	Brown, bluish-gray or black, 15% non-pigmented	Brown or black	Variable
Size (mean diameter)	<1 cm	0.5cm	Several centimeters	4 cm
Shape	Flat, solitary & well-circumscribed	Well-demarcated but elevated	Flat or slightly raised	Asymmetric with irregular outline
Commonly occurred site	Lip & gingiva	Palate	Buccal mucosa	Hard palate & maxillary gingiva
Causative factor	Melanin deposition	Proliferation of melanocytes	Proliferation of keratinocytes & melanocytes	Uncontrolled growth of melanocytes
Histopathologic features	Melanin accumulation without an increase in melanocytes.	Polygonal & epithelioid nevus cells in the superficial. Cytoplasm transparent to light stained.	Many dendritic melanocytes, processes containing melanin & melanophagocytes in all strata of epithelium.	Large, vesicular nucleus & prominent nucleoli. Aggregated into sheets or alveolar groups. Neurotropic or desmoplastic configurations.

### Melanotic Macule of the Oral Mucosa

Melanotic macules are one of the most common melanocytic lesions (48, 49) and lentigo simplex is the term used to describe a group of small and round macules (50). The color of macules varies from gray to brown to black. The diversity of pigmentation is thought to be associated with the ratio of eumelanin and pheomelanin (23, 24). Macules are usually regarded as benign lesions since the causative factor of macules is melanin deposition and no Ki-67 positive melanocytes are observed (51, 52). Hence, the diameter of the pigmented lesions is usually less than 1 cm, and their morphology is flat, solitary, and well-circumscribed (**Figure 1A**). From histological examinations, the basal cell layer of benign macules is exhibited with uniform melanin accumulation without an increase in the density of melanocytes or the presence of nevus (**Figures 1E, F**). These lesions are asymptomatic and no malignant transformation is reported at this stage. The most frequently observed site for macules in the oral cavity is the vermillion border of the lip at the rate of 30% followed by the gingiva and alveolar ridge (23%), and the buccal (16%) or labial mucosa (9%) (24). Interestingly, the hard palate, which is one of the most common locations for OMM has less chance for macule occurrence (7%) (24). Although there is no evidence that melanotic macules are directly associated with the eventual diagnosis of MM in oral mucosa, some published case reports have recorded the transformation of benign macules to malignant OMM after years of diagnosis (53–55), suggesting the malignant potential of some macular lesions to be considered as precursor lesions. As Ki-67 staining is not routinely requested in the diagnosis of melanotic macules, it is unclear what percentage of lesions contain proliferating melanocytes and may possess malignancy potentiality.

### Oral Mucosal Nevus

Oral nevi are much less common than their counterparts on the skin and their prevalence is about 0.1% in the general population (24). Subepithelial lesions are the most common oral mucosal nevi (55%), followed by blue nevi in submucosal–mucosal junction (**Figures 1I, J**) (36%), and junctional nevi are the least frequent ones (3%) (7, 56). According to the histologic location of melanocytes, oral nevi can be divided into three categories: junctional nevi (**Figures 1K, L**) at the tip of the widened and elongated epithelial spikes; compound nevi arranged in nests and belts in the lamina propria; and subepithelial nevi (**Figures 1B, G, H**) entirely in the subepithelial connective tissue. The formation of nevi in oral mucosa results from the proliferation of melanocytes along with the epithelial basal cell layer, but most are relatively small with a mean diameter of 0.5cm. Similar to macules, nevi harbor clear borders. However, instead of being flat, more than 50% of nevi are elevated pigmented lesions. In addition, about 15% of oral nevi are non-pigmented and the mechanism behind the lack of pigmentation remains unclear (24). From the histopathological point of view, the appearance of nevus cells along epithelial spikes is polygonal and epithelioid. Typical nevus cells have uniformly round or oval nuclei and contain sparse, uniform, and small melanin granules in the cytoplasm. As for nevus cells in the deeper subepithelial tissue, they become smaller with less cytoplasm and dense and deeply stained nucleus-like lymphocytes.

Although there is a lack of case reports unequivocally documenting the transformation of benign nevi to malignant tumors in the oral cavity, the risk of malignancy in some oral nevi cannot be excluded. The deficiency of case reports is partially due to the rare individuals with congenital or acquired nevi and short follow-up periods of objects (57). A clinicopathologic analysis shows that five out of seven OMM patients have junctional nevi, therefore some clinicians recommend a complete excisional biopsy to rule out early OMM for individuals with junctional nevi (58). In addition, nowadays the classification of nevi is mainly based on their histologic positions and lacks criteria based on the degree of malignancy. Only the appearance of dysplastic nevi is considered as an increased risk of melanoma (59–61). Dysplastic nevi are usually larger than normal nevi with macular or popular components and ill-defined borders (62). The current diagnosis of dysplastic nevi mainly depends on their architectural disorder rather than specific biomarkers, which heavily relies on the experience of pathologists and causes a relatively high rate of misdiagnosis. Hence, more refined diagnostic criteria and more sensitive biomarkers are needed clinically to find potential precancerous nevi.

### Melanoacanthoma

Melanoacanthoma is a rare form of benign melanotic lesion characterized by benign proliferation of both keratinocytes and melanocytes (24, 63). Microscopic examination can detect the hyperplastic keratinocytes, while positive immunostainings of HMB-45 and S-100 prove the presence of melanocytes abnormal accumulation (64). Compared with macule and nevus, this benign entity is much rarer but may mimic OMM due to its rapid increase in size with diameters of several centimeters being reached in just a few weeks. The lesion is usually flat or slightly raised and most commonly occurs on the buccal mucosa. Histopathologic examination shows many dendritic melanocytes and processes containing melanin in all strata of epithelium. Besides, melanophagocytes, mild lymphocyte infiltration, as well as vasodilation are seen in the lamina proporia. Similar to other benign lesions, once the melanoacanthoma is diagnosed, the site is usually just monitored, as these lesions are highly likely to regress within 2 to 6 months after biopsy (65, 66). However, if a melanoacanthoma enlarges in a very short period of time, it may indicate a sign of malignancy (67). A more comprehensive understanding of what drives mucosal melanocyte proliferation as well as the regression in melanoacanthoma, and how the fast-growing melanoacanthoma transforms into MM, is needed.

Oral macule, nevus, and melanoacanthoma are usually diagnosed as benign lesions, but periodical physical examinations and biopsies of those melanocytic lesions are still recommended because approximately one-third of OMM patients are found to present benign pigmented lesions prior to the emergence of the malignant state (67–69). Additionally, our collaborating clinicians and pathologists at Shanghai Ninth People’s Hospital have observed the development of hyperpigmentations adjacent to OMM in a majority of patients and they suspect that tumors expand through those *de novo* pigmented lesions (**Figures 1D**). Based on the above findings, we propose that a subgroup of benign lesions, especially macules, possess the potential to transform to OMM. If true, identification of biomarkers for those cancer-predisposing lesions coupled with more radical surgical excision may improve the outcome of patients. To achieve this goal, a thorough genetic evolution study sequencing not only malignant MMs but also benign lesions and suspected premalignant lesions is needed. Assessment of the genetic evolution of benign and malignant MM subtypes may reveal markers of increased risk of malignant transformation to aid in early diagnosis and clinical management.

## Mucosal Melanoma

OMM is one of the most frequent and well-studied MM subtypes. The preferred site for OMM (**Figure 1D**) is the keratinized mucosa, including the hard palate and maxillary gingiva where the masticatory stress is focused (70). Symptoms include pain, ulceration, bleeding, loose teeth, bone erosion, etc. (71, 72). The MM shows variable color from black to red or white accompanied with asymmetric and irregular morphology (73). Contrasting from CM, which is commonly diagnosed in the radial growth phase, OMM is usually first identified in a vertical growth phase with 30% of lesions at an invasive stage, and 55% of lesions at a combined invasive and *in situ* stage (7). Also different from CM, MM lacks a clear classification system for subtypes of lesions. Based on the histopathologic patterns and levels of solar damage, there are several different categorization methods for CM (74), whereas the subclassification of MM remains controversial. Currently, it is simply divided into MM *in situ*, invasive MM, and MM with a mixed pattern. The observing surface architecture of MM ranges from macular to ulcerated and nodular (75). Lentigo maligna melanoma (**Figure 1C**) is regarded as one form of OMM *in situ* as it shares similar histopathological traits as typical OMM *in situ* (**Figures 1M, N**). From the microscopic perspective, OMM consists of diverse morphological melanocytes including epithelioid, spindle, and plasmacytoid, which typically have a large, vesicular nucleus with prominent nucleoli (**Figures 1O, P**). They are usually aggregated into sheets or alveolar groups and less commonly neurotropic or desmoplastic configurations are observed. Most of the tumors contain melanin, while only a small proportion is amelanotic (76). As for immunohistochemical features of OMM, there is no single immunohistochemical marker that invariantly identifies all OMM. A variable expression of S-100, Melan-A, MITF, tyrosinase, and HMB-45 has been reported (27, 28, 49, 76). SOX 10 is a new marker, showing high sensitivity (positive in 88-100% of OMM cases) but moderate specificity in MM (77, 78). Hence, identification of biomarkers for OMM with better test characteristics of needed to achieve a consistent accurate diagnosis of MM and its initial lesions.

## Mutations and Signaling Pathway Dependency in MM

**Figure 2:**
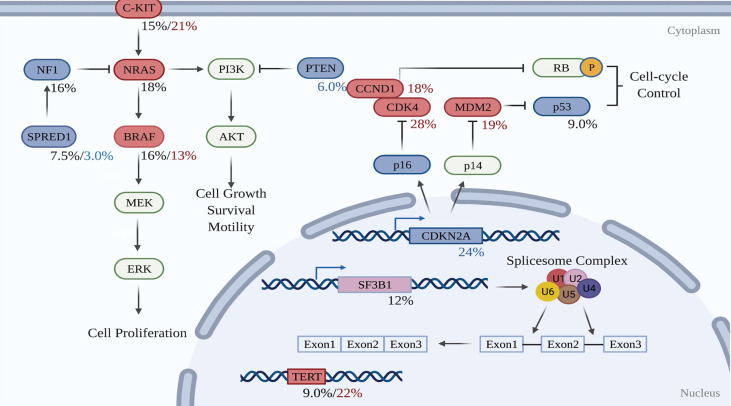
Molecular pathways involved in the development of mucosal melanoma. Red-filled rectangles indicate genes experiencing activating mutation or amplification, while blue-filled rectangles genes undoing suppressing mutation or deletion. Black figures suggest mutation rates, whereas red and blue percentages are respectively amplification and deletion proportions in the test cohort. Created with BioRender.com (2021). *Retrieved from* https://app.biorender.com/biorender-templates.

To date, whole-genome sequencing (WGS) and whole-exome sequencing (WES) has revealed the genomic profile of MM and pinpointed reoccurring aberrant genes that potentially drive the evolution of melanocytes to malignant tumors in the mucosal membrane. In contrast to CM, MM harbors a low single nucleotide mutation burden, but a high number of chromosomal structural variants (16, 79). *BRAF* and *NRAS* mutations, which are widely present in CM, are less frequent in MM (16, 79). Instead, activating mutations in *SF3B1* and *KIT*, loss of *CDKN2A, PTEN*, or *SPRED1*, as well as amplification of *CDK4, TERT, KIT, MDM2*, or *CCND1*, are more common in MM (16). **Table 2** compares the genetic profile between CM and MM. The data for altered genes in CM are average from Akbani’s and Hayward’s papers (17, 79), while the figures for MM are obtained from Newell’s paper (16). **Figure 2** summarizes the frequency of alterations in possible driver genes based upon WGS data of 67 frozen tumors (16). Those mutated genes correspond to specific cellular pathways that are potentially highly dependent on the initiation and progression of MM, providing potentially effective targets for combined treatment in the clinic.

**Table 2:** Comparison of genetic profiles MM and CM.

Cellular pathway	Gene	CM	MM
C-KIT pathway	*KIT*	3.7_mut_; 4.2%_amp_ (17, 79)	15%_mut_; 21%_amp_ (16)
MAPK pathway	*NRAS*	29%_mut_ (17, 79)	18%_mut_ (16)
	*BRAF*	51%_mut_ (17, 79)	16%_mut_; 13%_amp_ (16)
	*NF1*	15%_mut_ (17, 79)	16%_mut_ (16)
	*SPRED1*	rare	7.5%_mut_; 3.0%_del_ (16)
PI3K pathway	*PTEN*	9.0%_mut_; 12%_del_ (17, 79)	6.0%_del_ (16)
Splicesome pathway	*SF3B1*	6.4%_mut_ (17, 79)	12%_mut_ (16)
Cell cycle pathway	*TP53*	16%_mut_ (17, 79)	9.0%_mut_ (16)
	*CDK4*	4.0%_amp_ (17, 79)	28%_amp_ (16)
	*CCND1*	5.5%_amp_ (17, 79)	18%_amp_ (16)
	*MDM2*	3.5%_amp_ (17, 79)	19%_amp_ (16)
	*CDKN2A*	16%_mut_;44%_del_ (17, 79)	24%_del_ (16)
Telomere maintenance	*TERT* promoter	72%_mut_ (17, 79)	9.0%_mut_ (16)
	*TERT*	8.2%_amp_ (17, 79)	22%_amp_ (16)

Mut, mutation; Amp, amplification; Del, deletion.

### KIT Signaling Pathway

C-KIT is a receptor tyrosine kinase located on the membrane of various cell types. The stimulation of the C-KIT receptor by its extracellular ligand leads to downstream activation of the MAPK and PI3K signaling cascades that play an important role in proliferation, survival, and motility of melanoma cells (80). There is a high prevalence of *KIT* gain-of-function alterations including missense mutation and copy number amplification in patients with MM at rates of 15% and 21% respectively (16), while the corresponding figures in CM are only 3.7% and 4.2% separately (17, 79). MMs with *KIT* mutations presumably affect the function of juxta-membrane autoinhibitory domain (JMD) (W557R, N566D, V559A, V559D, V560D, V569G, P573L, L576P, K642E) and tyrosine kinase domains (D816H, D820Y, A829P, N822K), causing constitutive activation of C-KIT-regulated pathways (81–84). Among the aforementioned mutations, K642E is the most frequently observed in MM. Although codon 642 is located out of the JMD, amino acid aberrations in this position are thought to destabilize the JMD through amino acid interactions (81). Besides WGS and WES data, immunohistochemistry images show increased protein expression of C-KIT in all *in situ* MMs and nearly 90% of invasive tissues in a cohort of 18 cases (85), indicating the strengthening of the C-KIT signaling pathway. Compared to CM, the gain-of-function alterations of *KIT* are more common not only in MM but also in acral melanoma (AM) (86). Although the mechanism explaining why C-KIT is pathogenetically important in sun-protected melanomas remains poorly understood, it does not prevent the protein from being a potentially effective therapeutic target and a series of C-KIT inhibitors are currently under pre-clinical and clinical investigations (82, 87–89).

### RAS-RAF-MEK-ERK MAP Kinases Pathway

Stimulation of C-KIT or other receptor tyrosine kinase on the cellular membrane by extracellular growth factors provokes downstream activation of RAS and RAF kinases followed by phosphorylation of MEK and ERK, leading to activation of the MAPK pathway implicated in the regulation of cell proliferation, differentiation, and survival (90). NRAS and BRAF both play a part in the MAPK pathway, which are thought to contribute to melanoma development.

*NRAS* activating mutations are prevalent in CM at 29% (17, 79), while the mutation frequency in MM is only 18% (16). Furthermore, nearly 90% of *NRAS* missense mutations occur at codon 61 in CM, compared to 54% for MM. The remaining 46% of mutations are located at codon 12 and codon 13 (91). Compared with *NRAS* activating mutations at positions 12 and 13, *NRAS* Q61 mutations exert a stronger activating effect on the MAPK pathway since codon 61 is the catalytic residue for GTP hydrolysis and Q61 mutation impedes the return of RAS to an inactive GDP-bound state (92). For both CM and MM, Q61R and Q61K are the most commonly detected amino acid transitions at codon 61. Similarly, the most common mutations for both types of melanoma at codon 13 are G13D and G13R, although G13R is predominant in CM and G13D in MM (93).

BRAF is a serine-threonine kinase involved in the MAPK signaling pathway. Over 50% of CM cases report activating mutation of the *BRAF* gene (17, 79), while merely 16% of MMs experience the same alteration (16). In addition to harboring common active mutations, *BRAF* is distinct from *NRAS* in MM in that the locus undergoes amplification in 13% of cases (16). Mutations in the *BRAF* gene are missense mutations and they most frequently occur at codon 600, the activating loop, where amino acids change from valine to glutamic acid (V600E) (93). Besides the activation loop (A-loop), the second most common site for amino acid substitutions is the GSGSFG phosphate-binding loop (P-loop) at residues 464-469 (94). The activity of BRAF kinase is regulated by the interaction formed between A-loop and P-loop, thus mutations in either A-loop or P-loop disrupt the interaction and cause hyperactivation of the kinase (93, 95). In CM, more than 90% of mutations are present on the V600 codon, whereas in MM only 63% are V600 mutations, with the remainder (37%) on the G469, D594, and K601 codons (91, 93). In other words, MM not only has far less frequent *BRAF* missense mutations but also has more diverse locations for *BRAF* mutations as compared to CM. However, similar to CM there is nearly no coincidence of *NRAS* and *BRAF* missense mutations, suggesting functionally redundant *NRAS* and *BRAF* mutations in MM despite more variable mutational locations.

Although the contribution of mutant *NRAS* and *BRAF* to MM progression appears to be less common than melanoma of the skin, more components in the MAPK pathway of MM tend to undergo mutations or copy number changes, rendering inhibition of MAPK signaling transduction more challenging. The *NF1* gene, for example, encodes neurofibromin 1 protein, a negative regulator of Ras proteins, and can lose its function in both CM and MM. *NF1* mutation rates are 16% in MM (16) and 15% in CM (17, 79), suggesting *NF1* plays a pivotal role in the biology of both types of melanoma. Loss of *NF1* is associated with sustained activation of Ras proteins, leading to hyperactivation of MAPK and PI3K-AKT intracellular signaling pathway that evokes melanogenesis. Similar to CM, *NF1* suppression is significantly enriched in tumors lacking either *BRAF* or *NRAS* mutations (16, 96). However, in melanomas harboring both *BRAF* and *NF1* mutations, it is more likely that tumors can escape from MAPK inhibiting therapy (97, 98). Meanwhile, *NF1* is significantly co-mutated with *KIT* in 32% of MMs, whereas the co-occurrence level in CM is merely 4% (99), which indicates that the MAPK cascade is upregulated in MM not only by the single protein in the cytoplasm but also by the assistance of C-KIT receptor on the cell membrane.

*SPRED1* is another potential driver gene for MM. SPRED1, sprout-related, EVH1 domain containing protein 1, is a tumor suppressor. SPRED1 facilitates the localization of NF1 to the plasma membrane where it suppresses RAS signaling (6). Therefore, the loss of SPRED1 function leads to the activation of MAPK pathway signaling transduction. 7.5% of MM have *SPRED1* inactivating mutations and 12% undergo *SPRED1* copy number loss (16), whereas *SPRED1* alterations are insignificant in CM (17, 79). In MM, *SPRED1* loss rarely co-occurs with *BRAF* mutations, *NRAS* mutations, or *NF1* inactivation mutations (6), indicating those alterations play similar roles in activating MAPK pathway signaling in MM. Analogous to *NF1*, around 30% of MM cases with *SPRED1* inactivation simultaneously exhibit *KIT* alterations, suggesting that *SPRED1* inactivation may be in collaboration with other oncogenic events to stimulate tumor development (100). Based on the pattern of mutually exclusive occurrence of *NF1* and *SPRED1* and their respective tendency to alter simultaneously with *KIT*, it is reasonable to speculate that NF1 and SPRED1 loss function similarly in MM. In addition, it has been proposed that the reduced sensitivity and drug resistance to KIT inhibitors partially result from the hyperactivation of MAPK caused by the loss of *SPRED1* – a model verified in human melanoma cell lines and *in vivo* zebrafish model (6, 101), but presently untested in mouse models and patient samples.

### PI3K-AKT-mTOR Pathway

The PI3K-AKT-mTOR pathway is another frequently activated oncogenic signaling cascade in MM, which is verified by elevated AKT phosphorylation through immunohistochemical staining (102, 103). The aforementioned abnormal *KIT*, *NRAS*, *NF1*, and *SPRED1* genes are able to not only activate the MAPK signaling cascade but also dysregulate the PI3K-AKT-mTOR pathway. Additionally, the PI3K pathway is stimulated by suppression of a negative regulator, phosphatase and tensin homologue (PTEN) (104). Compared with 12% of *PTEN* loss in CM where deletions and mutations both account for the changes (17, 79), *PTEN* is deeply deleted in merely 6.0% of MM cases and has hardly any mutations (16) Furthermore, there is rare co-occurrence of deleted *PTEN* and amplified *KIT* that possibly implies that loss of *PTEN* or gain of *KIT* are redundant for the activation of the PI3K pathway in MM. This is also supported by the fact that besides *KIT* and *PTEN*, mutations in PI3K and AKT homologous of PI3K-AKT-mTOR are scarce (4.8% *PIK3CA*, 3.8% *PIK3CG*, and 4.8% *AKT3*) *(* 105). It has been reported that silencing of PTEN cooperates with activated AKT to promote metastasis of melanoma (102, 103, 106, 107), but MM does not show a weaker performance in metastasis than CM in the clinic possibly because there are other gene alterations in the PI3K pathway promoting invasiveness of tumor (108). In a cohort of 91 MM patients, 18% of cases show TSC1 loss-of-function mutations which plays a suppressive role in cellular proliferation initiated by mTOR (109) and a similar result also showed in a recently published meta-analysis review of MM (105). Due to the limited sample size, the alteration levels for TSC need to be further verified. Taking into account the frequency of alterations in known genes involved in PI3K-AKT-mTOR pathway activation, applying PI3K pathway blockers could possibly be an effective target strategy in MM patients.

### The Spliceosome Pathway

The spliceosome complex is responsible for the removal of introns from precursor mRNA and the ligation of exons to form mature mRNA. SF3B1 (splicing factor 3b subunit 1) is the largest and core component of the U2 small nuclear ribonucleoprotein (snRNP) and thus *SF3B1* mutations directly cause aberrant gene transcripts which eventually lead to mRNA degradation or abnormal protein function or protein decay (110, 111). *SF3B1* mutations have been reported in 12% of MM (16), while a very small portion of tested CM patients harbor similar alterations (17, 79, 112). Despite their notable absence in CM, alterations in the *SF3B1* gene are not unique to MM - similar mutations have also been detected in uveal melanoma, breast cancer, myelodysplastic syndromes, and chronic lymphocytic leukemia, including mutation hotspots such as codon 700, 622, 625, 662, and 666 (99, 113). To be more specific, *SF3B1* mutations at codon 625 are predominately associated with mucosal and uveal melanoma, while alterations at codon 700 are present across myeloid leukemia and chronic lymphocytic leukemia (6), implying disparate SF3B1 mutational preference that is possibly related to distinct etiology. Although *SF3B1* mutations are widely present in MM, it is poorly understood which genes alternatively spliced by mutant *SF3B1* drive malignant transformation. It has been discovered that *BRD9, PPP2R5A*, and *DVL2*, are candidate genes for alternative splicing in *SF3B1 K700*-mutant chronic lymphocytic leukemia (114–116), while *ABCC5*, *UQCC*, and *CRNDE* are possible targets in three uveal melanoma cases mixed with R625 and K700 mutations (117). Hence, due to the variability of SF3B1 mutations in solid and hematologic cancers, experiments that query the consequence of the *SF3B1 R625* mutation in mucosal melanocytes are needed to understand mis-spliced targets and tumorigenic oncogenic mechanisms related to SF3B1 mutations in MM.

Intriguingly, although SF3B1 does not possess a direct role in MAPK pathway signal transduction, there is little overlap between tumors with MAPK pathway mutations and *SF3B1*-mutated tumors, suggesting that SF3B1 mutations possibly lead to splicing variations of specific genes that can lead to MAPK activation (16). Meanwhile, the MAPK pathway can also regulate splicesome activity. It is reported that activation of the MEK-ERK pathway by Golgi stress enhances the activity of ETS transcriptional factors that have the capacity to regulate the expression of splicesome components, resulting in a switch of MCL1 protein function through different splicing (118). This study indicates that dysregulation of some downstream effectors by the MAPK pathway is able to lead to splicing aberrations. The mechanisms connecting the splicing alternations to signal transduction remain enigmatic. Mutant K700E *SF3B1* causes loss of function of phosphatase PP2A, followed by the phosphorylation changes related to signaling cascade (115), thereby providing a potential link between an alternative splicing and signaling pathways. Further investigations are needed to elucidate the specific mis-spliced genes and proteins directly influenced by *SF3B1* mutations together with the crosstalk between SF3B1 and MAPK pathway in tumorigenesis of MM, and the findings may provide a new perspective for targeted therapy.

### Cell Cycle Pathway

The abrogation of cell cycle checkpoint and apoptosis regulators is widely present in melanoma (119), including *CDKN2A* loss and *CDK4/6* or *CCND1* amplification. The *CDKN2A* locus encodes two distinct tumor suppressors, p16^INK4A^ and p14^ARF^. The p16^INK4A^ protein suppresses the forward progression of the cell cycle by inhibiting CDK4 or CDK6. The CDK4/6/CCND1 complex phosphorylates and inhibits the retinoblastoma (Rb), which leads to E2F1 transcription activation and G1-S phase cell cycle transmission (120). The other CDKN2A transcript, p14^ARF^, functions, at least in part, by blocking MDM2 ubiquitylation mediated TP53 degradation, which permits apoptosis escape (121). In MM patients, 24% of tumors exhibit copy number loss of *CDKN2A (* 16). Additionally, *CDK4*, *CCND1*, and *MDM2* are amplified in 28%, 18%, and 19% of samples respectively, and *TP53* mutations occur in 9.0% of MMs (16). Most of these cell cycle components are also commonly disrupted in CM. However, although CM has a much higher *CDKN2A* loss than MM, MM tends to show a greater frequency of *CCND1* and *CDK4* amplification (120, 122), implying CDK4 blocking agents may achieve a desired anti-tumor effect on MM. Intriguingly, MM cells without mutations in *BRAF* or *NRAS* mutations tend to exhibit *CCND1* or *CDK4* amplification (122), suggesting that copy number variations of cell cycle regulatory genes act as an alternative driver and can substitute for *BRAF* or *NRAS* mediated proliferation signaling pathway activation.

### Telomere Maintenance

Telomerase reverse transcriptase, encoded by the gene *TERT*, is the catalytic subunit of the enzyme telomerase, responsible for lengthening telomeres at the end of chromatin (123, 124). Thus, overexpression of TERT confers the potential of cells to become immortal (125), which is one of the hallmarks of cancer. *TERT* promoter mutations and *TERT* amplifications are common genetic events in the early stages of melanoma of the skin (8). For CM, more than two-thirds of tumors exhibit *TERT* promoter mutation, and only a minority of malignancies present copy number amplification (17, 79). As a comparison, the frequency of *TERT* activating alteration in MM declines to 30%, and most of them are copy number gain rather than promoter mutations (16). As for why CM and MM present distinct mechanisms of TERT activation, most *TERT* promoter mutations in CM are C>T mutations or CC>TT di-pyrimidine mutations (126, 127), suggesting that *TERT* promoter mutations are induced by UV radiation which partially explains why these mutations are rare in sun-shielded MM. Apart from *TERT*, the gene *ATRX* is also involved in telomere maintenance. *ATRX* is associated with alternative lengthening of telomeres as an additional mechanism for telomere maintenance in tumors lacking *TERT* promoter mutations (128). Despite rare samples associated with *ATRX* alterations in CM, nonsense mutation and frameshift of *ATRX* are detected in 11.9% of MMs (16) implying that *ATRX* is responsible for telomere extension in MM as well. However, the altering level of *ATRX* needs to be further tested in a larger cohort since the gene are not significantly mutated in other sequencing results except for Newell’s study.

Although CM exhibits a much higher frequency of TERT activation than MM, there is no statistically significant difference in telomere length among CM and MM, and both of them even undergo telomere shortening (8, 79, 129). Those intriguing findings remind us that aberrant *TERT* might have tumorigenic impacts in melanocytes other than telomere lengthening. It is reported that human TERT (hTERT) is equipped with a telomere protective function independent of its canonical catalytic activity (130). Overexpression of hTERT in melanoma is able to produce a protective complex on DNA damage that leads to the sustained proliferation capacity of cancer cells (130). In addition, phosphorylated TERT at a specific position by CDK1 has an RNA-dependent RNA polymerase (RdRP) activity (131). RdRP generates small interfering RNAs complementary to a tumor suppressor gene *FOXO4*, degrading mRNAs of *FOXO4*, reducing protein expression and consequently leading to tumor formation (131, 132). Taken together, these observations suggest potential differences in telomere maintenance mechanisms among different subtypes of melanoma.

## Progress in MM Target Therapy

When compared with CM, MM is typically detected at advanced stages, which renders the tumor challenging to treat. Surgical excision is predominately the first choice for MM (133–135). However, due to the lentiginous growth pattern, multifocal nature of MM, and limitations of the specific MM anatomic sites, it is extremely difficult for surgery to achieve wide negative margins, which leads to a high local relapse rate at 50%-90% (2). For unresectable and metastatic MM, targeted therapy and immunotherapy are constrained since MM is deficient in dominant MAPK activating mutations that can be targeted and is less responsive to immunotherapy (136). Therefore, to date, the first-line therapeutic modality for advanced MM remains chemotherapy despite limited efficacy (137).

While targeted therapies for MM are limited, multiple clinical trials targeting aberrant genes in MM are ongoing. Similar to CM, the MAPK cascade is hyperactivated by altered genes in MM including *NRAS*, *BRAF*, *NF1*, and *SPRED1*, thereby making inhibition of MAPK signal transduction a promising treatment strategy for MM patients. For the minority of MM patients with *BRAF* mutations, combined inhibition of BRAF and MEK is an attractive strategy because the combination therapy shows an impressive response rate at 76% and has a 5-year survival rate of 33% for *BRAFV600E/K* positive CM patients (138). Although there is no clinical trial underway specifically evaluating the safety and efficacy of combination therapy of BRAF inhibitor plus MEK inhibitor in MM, a retrospective study in Japan showed that MM/AM and CM exhibited similar response rates to combined BRAF and MEK suppression (64.3% vs 76.5%) (139), suggesting the potential efficiency of dual repression of BRAF and MEK in MM. For patients without *BRAF* mutations but with *NRAS*, *NF1*, or *SPRED1* alterations, targeting the downstream protein MEK is another strategy for MM patients. The safety and efficacy of MEK blocking agents in MM have been confirmed in an ongoing phase 2 study where 20% NRAS-mutated melanoma patients showed partial response to MEK inhibitor binimetinib with tolerated and manageable adverse events (140). However, for both monotherapy of MEK inhibitor and combined treatment of BRAF and MEK inhibitors, acquired resistance through reactivation of the MAPK pathway can restrict their therapeutic efficacy (141). To overcome this resistance, it is necessary to inhibit downstream proteins like ERK or develop new molecules targeting aberrant MAPK signaling. Meanwhile, besides independent suppression of MAPK pathway, MEK blockers have also been combined with mTOR1/2, AKT, or CDK4/6 inhibitors in preclinical models or in clinical trials of MM (142–144). Although therapeutic parameters do not significantly improve compared to the single MAPK inhibition, dual signaling pathway blocking still provides a new perspective for MM targeted therapy.

Interestingly, compared to CM, MM patients tend to harbor more activating mutations or amplifications in the receptor tyrosine kinase *KIT*, providing a rationale for targeting C-KIT. Imatinib, sunitinib, dasatinib, nilotinib, and masitinib are approved C-KIT inhibitors in different cancer types and their anti-cancer effects for MM are currently in the clinical research stage (145–148). Imatinib is the most widely investigated C-KIT inhibitor. In a recent trial of 78 melanoma patients harboring *KIT* alterations, the median overall survival for imatinib is 13.1 months and the objective response rate is 21.8% (149). Additionally, it has been discovered that C-KIT inhibitor imatinib harbors high efficacy against melanoma with *KIT* mutations, but not with *KIT* amplification only (54% vs 0% partial response) (148, 150). To be more specific, MMs with *KIT* mutations in exon 11 (L576P) and exon 13 (K642E) tend to have a better and longer response to C-KIT inhibitors than other mutations (84, 151). Despite the strong anti-tumor effect of C-KIT inhibitors, MM patients who respond to the inhibiting agents well at the beginning will frequently experience a brief period of disease response before developing resistance to KIT inhibitors that eventually leads to progressive disease (152, 153). The acquired resistance to KIT inhibitors is possibly conferred from pre-existing concomitant mutations in other oncogenes like *NRAS* or secondary *KIT* mutations during the use of drugs. For instance, secondary A829P *KIT* mutation renders melanoma cells resistant to imatinib but has no influence on nilotinib and dasatinib, while the T670I *KIT* mutation exhibits resistance to imatinib, nilotinib as well as dasatinib, but can still be suppressed by sunitinib (154). Considering the promising performance of C-KIT inhibitors in MM, now more efforts have been focused on the understanding of the acquired resistance mechanism and the development of new blocking agents to overcome resistance, offering hope for patients with advanced MM and limited treatment options.

In the future, targeted therapy could offer an alternative adjuvant therapy option for a group of patients based on their gene sequencing results. If actionable driver mutations are identified in an individual MM, targeted therapies for the driver genes or proteins could be utilized on a patient-by-patient basis. Until now there are only a few available targeted therapies for MM clinical trials: BRAF, MEK, CDK4/6 and, C-KIT inhibitors, with limited clinical use and efficacy. Therefore, it requires more efforts on developing other alternative targeting strategies based on mutated genes in MM such as splicesome complex components, telomerase, and DNA repair pathway. H3B-8800, for instance, is the blocker for splicing modulator of SF3B complex and it at present is in phase I study of myeloid cancers (155). Considering the MM specific SF3B1 hotspot mutation in R625, developing strategies that can specifically target R625 mutant SF3B1 might may achieve benefit MM patients with low side effects.

## Discussion

MM is a rare but aggressive malignancy. Due at least in part to delayed diagnosis at the advanced stage and the lack of efficient therapeutic strategies, this subtype of melanoma is associated with a worse prognosis than melanoma arising from the skin. In contrast to CM, the etiology, risk factors, and pathogenesis of MM are poorly understood, partially explaining the deficiency of effective treatment options and extremely poor prognosis. This review takes OMM as a model and attempts to identify commonalities in etiology, pathogenesis, mutation patterns, and corresponding pathway dependency. Besides cigarette smoking, denture irritation, and alcohol, chronic infections caused by microorganisms and mechanical stress generated by routine activities may have an impact on tumorigenesis in the mucosal membrane. However, the oral microflora is in a dynamic process of change and is influenced by many internal and external factors, including the host’s physical conditions, diet, and hygiene habits. A more comprehensive study investigating the relationship between flora and cancer, the selection of patients, sampling locations, and control settings will be needed.

Since there is limited knowledge about pre-MM lesions and a lack of corresponding molecular pathological biomarkers, early diagnosis, as well as early intervention becomes extremely challenging, leading to the short life expectancy in MM patients. Due to the unclear relationships between benign lesions and precursor lesions, histopathological information alone cannot thoroughly define and accurately discriminate them. It is reported that one patient died of OMM after 63 months of misdiagnosed premalignant atypical melanocytic hyperplasia as a benign lentigo simplex (50). Therefore, it is an urgent need to discover biomarkers for lesions with a greater tendency of malignant transformation. To achieve this goal, a thorough genomic and transcriptomic profiling of the evolutionary trajectories of MM starting from benign lesions and potential intermediate lesions is worth pursuit. Another strategy for studying cancer evolution is to establish transgenic mice that capture the evolution process of MM. However, unlike CM, there is a lack of animal models that can recapitulate the oncogenesis process accompanied with the accumulation of genetic alterations in MM. By stepwise introduction of *BRAF V600E* mutation, *CDKN2A* loss, *PTEN* loss and *mTOR* activation, CM precursor lesions followed by CM formation was observed in mice (156–159). Likewise, decoding the accumulative mutation pattern based on MM patient samples will pave the path to the generation of MM transgenic mice model, which not only contribute to understanding the pathogenesis of MM but also serve as functional tools to evaluate the efficacy of novel therapeutic modalities.

Recent sequencing studies have identified significant alterations in *NRAS, BRAF, NF1, KIT, SF3B1, TP53*, and *SPRED1*, informing potential targeted therapeutic strategies for MM (14–16, 160, 161). Firstly, MM patients have shown similar pathway dependency although with divergent mutation patterns. Compared to CM, fewer *NRAS*, *BRAF* mutations are seen in MM, but more *SF3B1* mutation and *KIT* alterations are found. Since targetable *BRAF* mutations are far less frequent in MM, target validation of other alterations in the MAPK pathway is needed. The sequencing results of 67 MMs show that mutations of *NRAS*, *BRAF*, *KIT*, and *SF3B1* are mutually exclusive, implying those mutations may converge on activating the MAPK pathways (16). Further studies about how *SF3B1* mutations are involved in MAPK pathway activation are needed. Secondly, MM has gained fewer genetic mutations for cell cycle regulators but more copy number changes than CM. While *CDKN2A* copy number loss is a frequently observed event in both CM and MM, MM presents more CDK4 and CCND1 amplifications, which makes targeting CDK4 promising in MM.

It is worth mentioning that MM has a much higher level of structure variation and chromosomal instability compared to CM. As a result, specific attention should be paid to targeting the chromosomal rearrangements. Targeting genes involved in DNA damage repair response including PARP, DNA-PKcs, ATR, ATM, CHK1, WEE1 might achieve unexpected clinical response in MM patients (162–164). Olaparib, for instance, is an FDA-approved inhibitor of the enzyme poly ADP ribose polymerase (PARP) which can efficiently kill BRCA mutant tumor cells, a successful synthetic lethality based targeting strategy used in breast cancers and ovarian cancers (165). Although MM rarely shows BRCA mutations, the significantly high level of structure variation indicates the deficiency of homologous recombination repair (HRR) capacity, which makes MM potentially responsive to PARP inhibition. Nevertheless, a more comprehensive understanding about the mutation signatures as well as signatures of chromosome structure variation in MM are needed. A more stringent validation of PARP inhibitor response in MM cell lines, PDX, and early clinical trials are supposed to conduct to better understand the pharmacological mechanism of drug response in MM.

Other than targeted therapy, immune checkpoint blocker (ICB) based immunotherapy has shown a strong anti-tumor effect on metastatic CM. Ipilimumab against cytotoxic T-lymphocyte antigen 4 (CTLA4), nivolumab and pembrolizumab against programmed death 1 (PD1) as well as atezolizumab against programmed death-ligand 1 (PD-L1) are approved by the FDA for the treatment for advanced melanoma either as monotherapy or combination therapy (166). The overall response rate (ORR) for CM patients to ipilimumab, nivolumab, pembrolizumab and atezolizumab is 12%, 40%, 33% and 30% respectively (167–169), while the combined treatment of ipilimumab with nivolumab significantly improves the ORR to 61% with median progression-free survival (PFS) of 11.5 months (170, 171). However, those ICBs do not exert a satisfactorily inhibitory impact on MM as they do on CM, showing the ORR to anti-CTLA4 or anti-PD1/PDL1 as the single agent from 7% to 35% (136, 172–174). Even the combination regimen of anti-CTLA4 (ipilimumab) and anti-PD1 (nivolumab) agents merely witness a slight increased ORR to 37% with PFS at 5.9 month (136, 175). The limited response to ICBs in MM is mainly because of low mutation burden and limited immune cell infiltration compared to CM (3, 176, 177). To further overcome unsatisfactory performance of ICBs in MM, combination of ICBs with different targeted therapy strategies has been tested in clinical trials. For example, a phase Ib trial using PD-1 antibody toripalimab and vascular endothelial growth factor receptors (VEGFR) inhibitor axitinib showed a dramatical improvement in ORR and PFS to 61% and 9.1 months separately (178, 179), although the safety and efficacy of this combination strategy needs to be further validated. Meanwhile, the combination of toripalimab and vorolanib which targets and inhibits multi-tyrosine kinase including VEGF and C-KIT are ongoing in MM trial (180).

In summary, both basic research and drug discoveries in CM have achieved enormous progress, whereas little is known about either how MM initiates or how to target MM. As a result, patients of MM are suffering from limited treatment options and undesirable response rates that lead to extremely poor prognoses. Here we summarize the current state of knowledge regarding initiation and progression of MM and the risk factors and treatment options for MM. In doing so, we highlight current gaps in our knowledge regarding MM progression, and propose important future research directions, includes studying the genetic evolution trajectory of MM from benign precursor lesions and evaluating new targeting strategies specifically for MM, such as targeting CDK4, SF3B1 or PARP, either as single agent or in combinations with ICBs. We hope these efforts will give more comprehensive knowledge about how MM initiates and progresses, provide more specific biomarkers for MM early diagnosis, offer more potentially effective treatment options for MM and, in the end, improve the life expectancy and quality for MM patients.

## Author Contributions

YM wrote the main body of the paper. HZ and RLJ revised the review. RX and XM provided significant intellectual contribution on clinical observations and pathological description of melanocytic lesions. HZ conceived and directed the idea of the manuscript. All authors contributed to the article and approved the submitted version.

## Conflict of Interest

The authors declare that the research was conducted in the absence of any commercial or financial relationships that could be construed as a potential conflict of interest.
